# Transcriptomic Profiling of Orbital Fat Tissue and Ocular Surface Wash in Active Thyroid Eye Disease Requiring Urgent Orbital Decompression

**DOI:** 10.1167/iovs.66.15.71

**Published:** 2025-12-29

**Authors:** Anna Petráčková, Jan Schovánek, Marta Karhanová, Jakub Savara, Romana Nesnadná, Eva Kriegová

**Affiliations:** 1 Department of Immunology, Faculty of Medicine and Dentistry, Palacky University and University Hospital Olomouc, Olomouc, Czech Republic; 2 Department of Internal Medicine III – Nephrology, Rheumatology, and Endocrinology, Faculty of Medicine and Dentistry, Palacky University and University Hospital Olomouc, Olomouc, Czech Republic; 3 Department of Ophthalmology, Palacky University and University Hospital Olomouc, Olomouc, Czech Republic; 4 Department of Computer Science, Faculty of Electrical Engineering and Computer Science, Technical University of Ostrava, Ostrava, Czech Republic

**Keywords:** thyroid-associated orbitopathy, thyroid eye disease (TED), disease activity, biomarkers, orbital decompression, RNA sequencing, (RNA-seq), fat tissue

## Abstract

**Purpose:**

Thyroid eye disease (TED) is an autoimmune disorder characterized by orbital inflammation and tissue remodeling. Although most patients with active TED respond to medical therapy, a subset develops sight-threatening complications which require urgent orbital decompression when conservative treatment fails. This study aimed to elucidate the molecular mechanisms underlying active, moderate-to-severe TED in patients requiring urgent orbital decompression during the active disease stage.

**Methods:**

Transcriptomic profiling was performed on retro-orbital fat samples from 13 patients and ocular surface wash samples from 9 patients undergoing urgent orbital decompression, as well as on samples from control subjects. Differential gene expression, pathway enrichment, cell-type composition, and drug-gene interactions were analyzed.

**Results:**

In retro-orbital fat, the majority of differentially expressed genes were upregulated, predominantly mapping to immune system, with a pronounced neutrophilic signature including degranulation and extracellular trap formation. Increased infiltration of neutrophils, B cells, and T cells was observed, whereas ocular surface wash samples exhibited a largely downregulated immune signature, reflecting compartment-specific immune responses. Expression of several transcripts from ocular surface wash correlated with patients’ disease activity, suggesting potential use as noninvasive biomarkers with *CD151*, *MAST4*, and *HPCAL1* genes correlating best. Drug-gene interaction analysis nominated JAK and BTK inhibitors as candidate therapeutics in TED.

**Conclusions:**

This study provides a unique molecular atlas of active, moderate-to-severe TED environment, uncovers the active role of neutrophils in TED pathogenesis, and identifies candidate therapeutic targets and noninvasive biomarkers that may inform future clinical strategies.

Thyroid eye disease (TED) is a potentially sight-threatening autoimmune ocular disorder linked to systemic thyroid diseases, most commonly Graves’ disease.^1^^,^^2^ TED is characterized by expansion of the extraocular muscles and orbital adipose tissue, which, depending on disease severity, may result in orbital disfigurement, diplopia, exposure keratopathy, or vision-threatening dysthyroid optic neuropathy (DON).^1^^,^^2^

TED is considered an autoimmune condition triggered by autoantibodies directed against shared thyroid-orbit antigens, particularly the thyroid-stimulating hormone receptor (TSHR) and/or the insulin-like growth factor-1 receptor (IGF-1R).^2^^,^^3^ Activation of the immune system, combined with the stimulatory effect of these autoantibodies on TSHR and IGF-1R, promotes orbital fibroblast proliferation, increased glycosaminoglycan synthesis (notably hyaluronic acid), adipogenesis, and fibrosis, thereby perpetuating tissue expansion and inflammation.^2^^,^^3^ Despite these insights, the exact pathogenesis of TED remains incompletely understood.^2^

Due to the limited understanding of its underlying mechanisms, effective treatment of TED remains a challenge.^2^ Glucocorticoids (GCs) currently represent the first-line therapy for moderate-to-severe active TED for most patients worldwide, aiming to suppress nonspecific inflammation. However, efficacy of immunosuppressive therapy with high-dose intravenous GC (IVGC) varies between 50% and 80% according to the published trials,^2^ and second-line treatment options are required for nonresponders. Whereas most patients with active TED do not require surgery, urgent orbital decompression is necessary when conservative treatment fails and when there is an immediate threat to vision due to DON.^4^^,^^5^

TED is variable in disease activity and severity, and available therapies are still inadequate. Therefore, there is a growing need for a deeper understanding of TED pathogenesis to identify novel pharmacological targets, as well as factors that contribute to TED onset, severity, and response to GC or immunotherapy.

In this study, we aimed to explore the molecular mechanisms underlying TED using transcriptomic analysis of retro-orbital fat tissue and cells obtained from ocular surface wash samples obtained from patients with active, moderate-to-severe TED who were treated with high-dose IVGC but did not respond and required urgent orbital decompression surgery due to optic neuropathy during the active phase of disease. Furthermore, we retrospectively characterized the cohort of patients with TED from a single tertiary center who required urgent orbital decompression during the active phase of the disease.

## Methods

### Study Cohort and Sample Collection

This real-world, single-center, prospective study consisted of patients with TED who were diagnosed and treated according to the European Group on Graves’ orbitopathy (EUGOGO) clinical guidelines^6^ at University Hospital Olomouc between October 2021 and September 2024. Demographic, clinical, and laboratory data related to TED were collected through a comprehensive review of medical records.

Orbital fat tissue samples were obtained from 13 patients with TED who underwent urgent orbital decompression (median age 54 = 40–72 years; male/female = 7/6) and from 5 control age-/gender-matched subjects who underwent upper eyelid blepharoplasty (median age = 54, 42–69 years; male/female = 3/2). Eyelid orbital fat is a reference orbital fat tissue control for TED studies. The collected tissues were frozen fresh on dry ice and stored at −80°C. Ocular surface wash samples were collected from nine patients with TED prior to decompression surgery (median age = 54, 40–69 years; male/female = 7/6) and from age-/gender-matched five control subjects (median age = 53, 42–68 years; male/female = 3/2). In the majority of patients (7/9) who agreed to participate in the ocular surface wash experiments, both eyes were affected. In all control subjects, the presence of autoimmune diseases, recent vaccinations or infections, and the use of immunosuppressive drugs were excluded based on a questionnaire administered during clinical examinations. The samples were obtained by trained medical personnel who gently dispensed 5 mL of sterile saline solution across the ocular surface and the runoff was collected in a sterile 50 mL tube. Samples were immediately centrifuged at 300×g for 7 minutes, and the cell pellets were stored at −80°C.

All patients and controls provided written informed consent for the usage of biological materials for the purpose of this study, which was conducted in accordance with the Helsinki Declaration and approved by the Ethics Committee of the University Hospital and Palacký University Olomouc (Ref. No. 49/23).

### Sample Preparation for RNA Sequencing and Data Analysis

Total RNA was isolated from pelleted cells from ocular surface wash samples using the RNAqueous-Micro Total RNA Isolation Kit (Invitrogen), and from fat tissue samples using the mirVana miRNA Isolation Kit with phenol (Invitrogen), following the manufacturers’ protocols. RNA quality and quantity (Supplementary Table S1) was assessed using the Agilent RNA 6000 Pico Chip (Agilent Technologies). Residual DNA contamination in these samples was removed by pretreatment with a double-strand specific DNase (ArcticZymes Technologies), according to the manufacturer's instructions. RNA sequencing (RNA-seq) libraries were prepared using the SMARTer Stranded Total RNA-Seq Kit version 3 – Pico Input Mammalian with Unique Molecular Identifiers (Takara Bio), which is particularly suitable for library preparation from total RNA of both high and low quality (RIN 2–10). In addition, this strand-specific kit utilizes random priming to synthesize cDNA from both polyadenylated and non-polyadenylated RNA, thereby ensuring the capture of the non-coding RNA fraction as well. Libraries were sequenced on the Illumina NovaSeq 6000 platform, targeting 50 million reads per sample. Library preparation and sequencing of all samples belonging to the same material type (fat tissue or ocular surface wash) were conducted in a single batch to avoid batch effects.

Quality control of RNA-seq reads were performed using FastQC version 0.11.9 (quality metrics provided in Supplementary Table S2). Reads were aligned to the human reference genome (GRCh38) using STAR version 2.7.9a. Read counting at transcript level was performed using Salmon version 1.10.1 relying on the Ensembl gene annotation file release 107. Resulting transcript quantification data were processed using the 3D RNA-seq analysis pipeline,^7^ which performed data pre-processing and differential expression analysis using limma. In the first step, read counts and transcripts per million (TPM) values were generated using the “lengthScaledTPM” method in tximport. Rarely expressed transcripts were then filtered out using counts per million (CPM) cutoff of 1 and a sample number cutoff of 1. Principal component analysis (PCA) was performed prior to differential expression analysis and revealed no outliers (Supplementary Fig. S1). Data normalization was performed using the Trimmed Mean of M-values (TMM) method. Differential expression analysis (pair-wise group comparison) was conducted using limma-voomWeights with thresholds set to an adjusted *P* value < 0.05 and an absolute log₂ fold change (|log₂FC|) set to 1, using the control group as the reference. For *P* value adjustment, multiple testing correction was applied using the Benjamini-Hochberg method. Raw gene read counts and TPM values are provided in Supplementary Table S3. Gene set enrichment analysis (GSEA) was conducted using Gene Ontology (GO), Kyoto Encyclopedia of Genes and Genomes (KEGG), and Reactome databases via ShinyGO version 0.82.^8^ Heatmaps were generated using Heatmapper.^9^ Estimation of cell type composition by computational deconvolution was performed using the EPIC tool with prebuilt reference^10^ and with data from single-nucleus RNA-seq.^11^ Cell-type specific and unique genes for T cells, B/plasma cells, neutrophils, and monocytes/macrophages were defined by a Tau specificity score > 0.80 using The Human Protein Atlas database (Supplementary Table S4).

Categorical variables between groups were compared using the chi-squared test, continuous variables by Mann-Whitney test and Spearman correlation, and time-to-event data by log rank test in Prism (GraphPad Software).

## Results

### Occurrence of Urgent Orbital Decompression – Real-World Evidence From Single Tertiary Center

**Figure 1.**
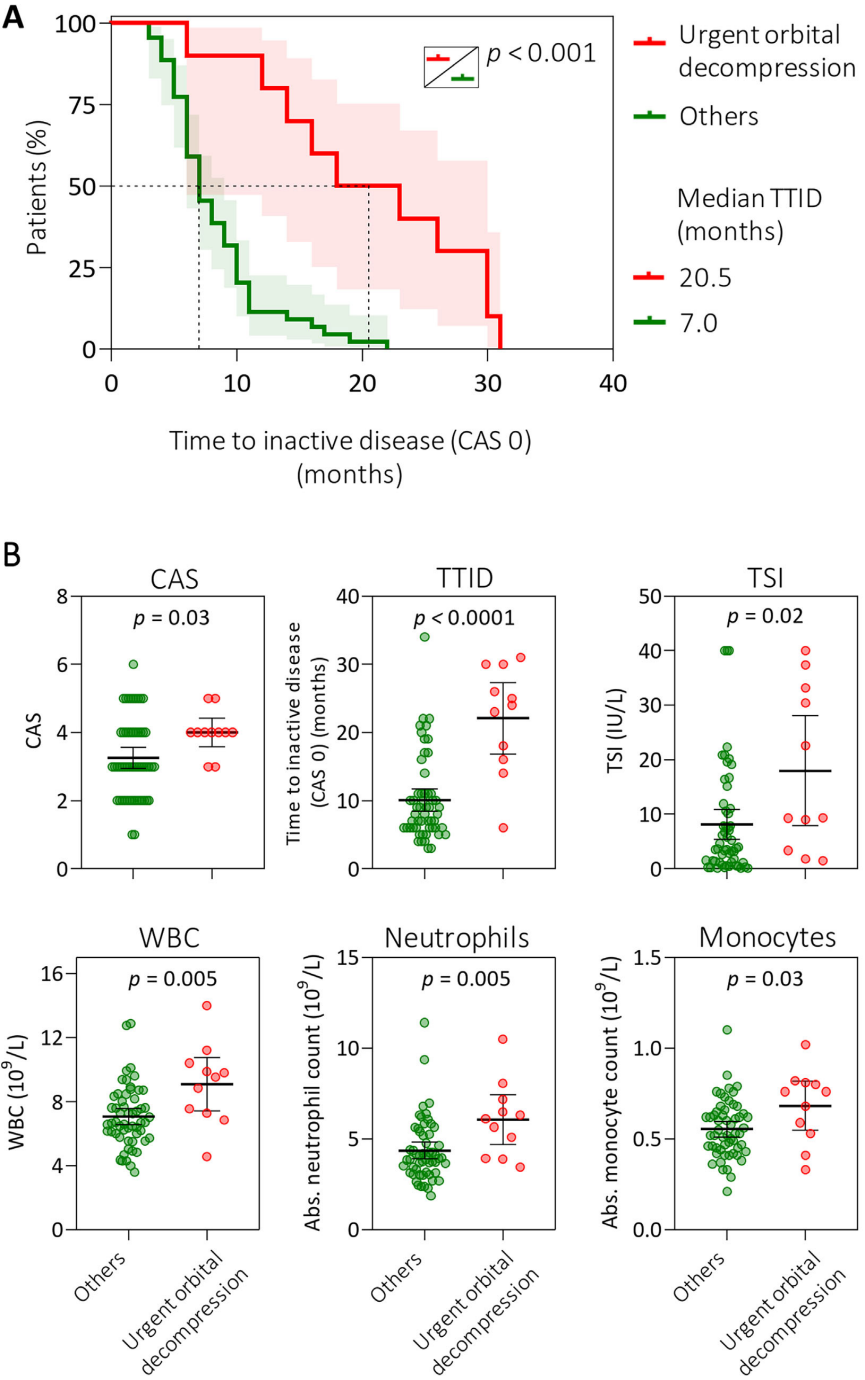
Baseline analysis of real-world TED cohort. (**A**) Time to inactive disease (TTID) in the TED cohort stratified into patients in whom urgent orbital decompression was required and other patients excluding steroid-resistant patients. (**B**) Differences in blood counts and antibody profile at diagnosis in the patient groups analyzed. Group means are indicated by *horizontal bars*; error bars indicate 95% CIs. CAS, clinical activity score; CI, confidence interval; TSI, thyroid-stimulating immunoglobulins; TTID, time to inactive disease; WBC, white blood cell count.

A total of 73 patients with active, moderate-to-severe TED requiring therapy were included in the study at a tertiary referral center, where patients with more severe presentations of TED are primarily encountered. The median follow-up was 12 months (range = 4–34 months) and clinical characteristics of the study cohort are in Supplementary Table S5. Urgent orbital decompression surgery was required in 20.6% (15/73) of patients, in 12 patients due to DON, and in 3 patients due to persistent severe exophthalmos unresponsive to IVGC treatment. In all cases, decompression surgery was performed during or after IVGC therapy. The median time from initiation of GC treatment to decompression surgery was 6.0 months (range = 1.0–13.0 months). In four of these patients, additional treatment with rituximab or tocilizumab was necessary to reduce the disease activity. The median clinical activity score (CAS) at diagnosis for patients who underwent urgent orbital decompression was 4 (range = 3–6) and the median time to inactive disease (TTID) which was defined as the time from therapy initiation to reaching CAS 0, was 20.5 months (6.0–31.0). Both CAS and TTID were higher in these patients compared with other patients in the cohort, who had a median CAS of 3 and a median TTID of 7.0 months (3.0–22.0; Fig. 1), excluding steroid-resistant patients. Steroid-resistant patients were defined as those exhibiting incomplete response to first-line IVGC therapy or those who experienced reactivation and additional medical intervention was needed.^12^

When looking at blood counts, the antibody (TSI, anti-Tg, and anti-TPO) and thyroid (FT4 and FT3) profiles at the time of diagnosis, patients who eventually required urgent decompression exhibited higher levels of TSI, white blood cell count, and absolute neutrophil and monocyte counts (see Fig. 1; Supplementary Table S5) compared with other patients, excluding steroid-resistant patients.

### Transcriptome Analysis of TED Retro-Orbital Fat Tissue

**Figure 2.**
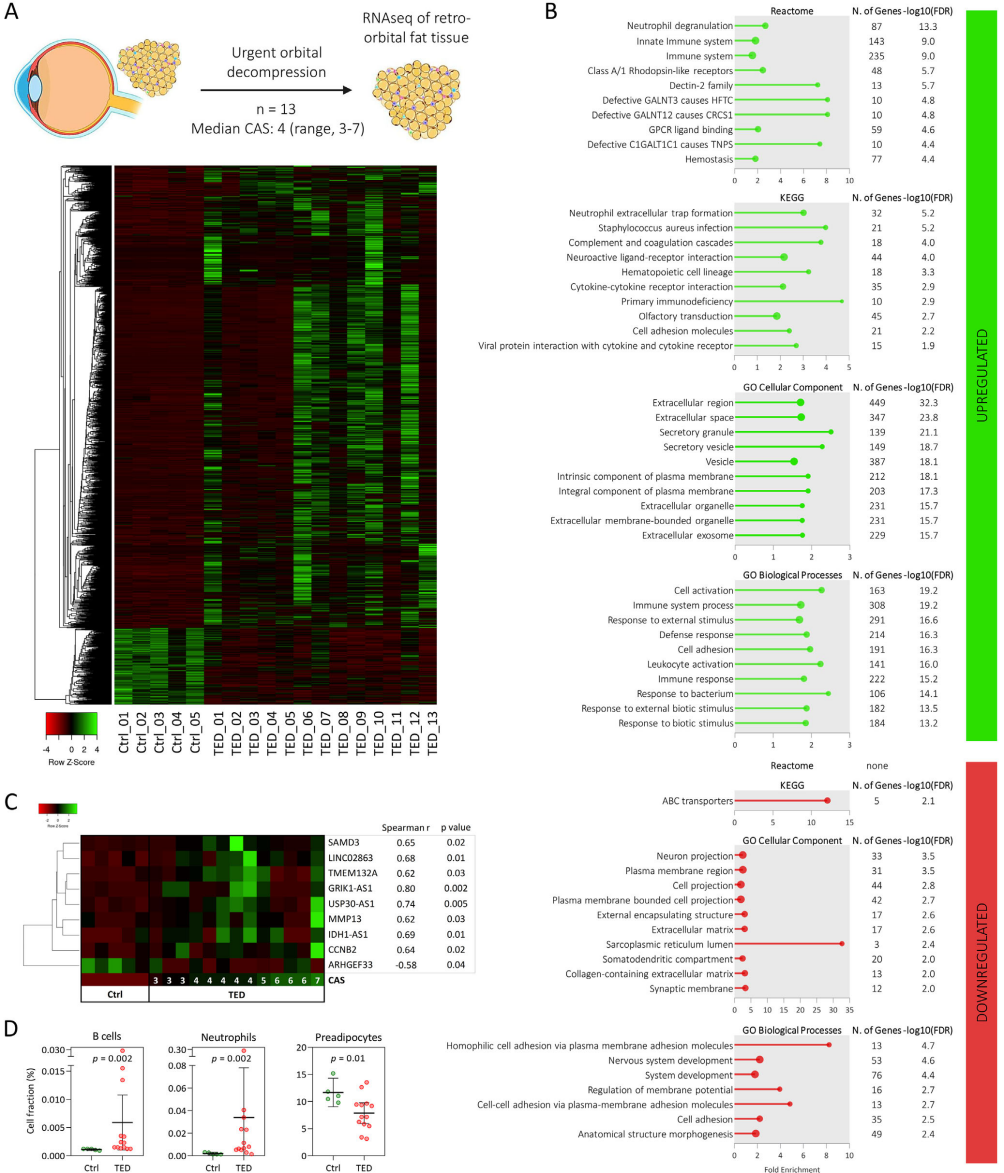
Transcriptomic profiling of retro-orbital fat tissue. (**A**) Heatmap of a total of 2458 differentially expressed genes (DEGs) in retro-orbital fat tissue obtained from 13 patients with TED (all with active disease except one with CAS 2), who underwent urgent orbital decompression, compared to 5 control samples. The diagram at the top of panel **A** was created with BioRender.com. (**B**) Gene set enrichment analysis of DEGs using Reactome, KEGG, and GO databases, showing either all or the top 10 significantly enriched pathways. (**C**) DEGs whose expression levels correlated with the patient's CAS at the time of surgery. (**D**) Estimated tissue composition of B cells, neutrophils, and preadipocytes by computational deconvolution of transcriptomic data. CAS, clinical activity score; GO, Gene Ontology; KEGG, Kyoto encyclopedia of genes and genomes; TED, thyroid eye disease.

Thirteen retro-orbital fat tissue samples were collected from 13 patients with TED who underwent urgent orbital decompression. In cases of bilateral decompression, only the tissue from the eye with the more severe clinical presentation was analyzed. All patients had active disease at the time of surgery (median CAS = 4, range = 3–7). To investigate gene expression changes in retro-orbital fat tissues associated with TED, RNA-seq was performed. A total of 2458 genes were found to be differentially expressed in TED compared with orbital fat tissues obtained from 5 control subjects undergoing blepharoplasty using the cutoff criteria of *P* adjusted value < 0.05 and |log_2_FC| set to 1 (Supplementary Fig. S2). Of detected differentially expressed genes (DEGs), 2109 (85.8%) were upregulated and 349 (14.2%) were downregulated (Fig. 2; see Supplementary Table S3).

**Figure 3.**
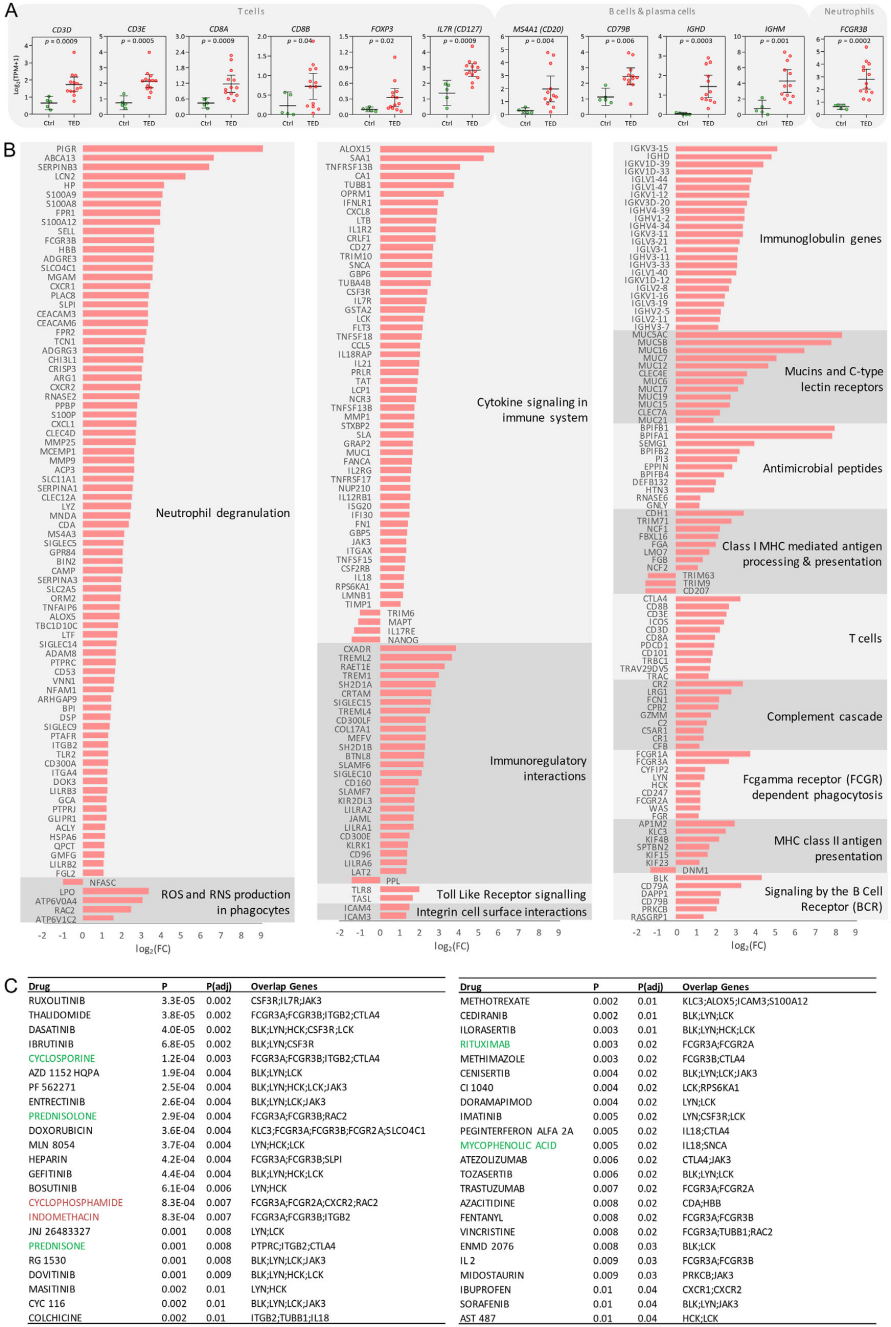
Transcriptomic profiling of retro-orbital fat tissue. (**A**) Expression of genes specific and unique to T cells, B/plasma cells and neutrophils. (**B**) Expression of 235 differentially expressed genes (DEGs) associated with immune system in retro-orbital fat tissue of patients with TED. (**C**) Therapeutic compounds identified by DGIdb that target proteins encoded by upregulated DEGs associated with immune system. Compounds *highlighted in green* are included in the EUGOGO clinical guidelines for the treatment of Graves’ orbitopathy^6^; compounds in *red* are used in the treatment of other autoimmune diseases.^14^^–^^16^ DGIdb, Drug Gene Interaction Database; EUGOGO, European Group on Graves’ orbitopathy.

The GSEA of the DEGs was performed using the Reactome, KEGG, and GO databases (see Fig. 2). Among the top enriched Reactome pathways from upregulated DEGs were neutrophil degranulation and immune system, with the largest subset of DEGs mapping to immune system-related pathways. The expression profile of 235 immune-related DEGs is shown in Figure 3, highlighting strong enrichment for genes associated with neutrophil degranulation. Additional immune-related pathways identified among the upregulated DEGs included cytokine signaling, immunoregulatory interactions. immunoglobulin genes, mucins and C-type lectin receptors, antimicrobial peptides, class I Major Histocompatibility Complex (MHC) mediated antigen processing and presentation, T cells, complement cascade, Fc gamma receptor dependent phagocytosis, MHC class II antigen presentation, signaling by the B cell receptor, integrin cell surface interactions, Toll Like Receptor signaling, and ROS and RNS production in phagocytes. In the KEGG pathway analysis, neutrophil extracellular trap formation and complement and coagulation cascades ranked among the top upregulated pathways. GO enrichment analysis of upregulated DEGs revealed an extracellular region and secretory granule among the top cellular components and cell activation and immune system process among the top biological processes.

We next assessed whether the proteins encoded by the upregulated immune-related genes (*P* < 0.01) could represent potential therapeutic targets using the Drug Gene Interaction Database (DGIdb).^13^ In total, 46 compounds targeting 29 immune-associated proteins were identified by DGIdb (see Fig. 3). Of these, five drugs (cyclosporine, prednisone, prednisolone, rituximab, and mycophenolic acid) are included in the EUGOGO clinical guidelines,^6^ and three drugs (cyclophosphamide, indomethacin, and methotrexate) are approved for other autoimmune diseases.^14^^–^^16^ Notably, the JAK inhibitor ruxolitinib and the BTK inhibitor ibrutinib emerged among the top novel drug candidates.

We also looked at gene expression of targets of approved targeted therapy or targets currently being evaluated in clinical trials: *TSHR*, *IGF1R*, *MS4A1* (CD20), *IL6*, *IL6R*, *IL11*, *IL11RA*, and *FCGRT* (FcRn).^17^ Of the genes analyzed, only upregulation of *MS4A1* (CD20) was detected and *IGF1R* was observed downregulated (Supplementary Fig. S3).

Of downregulated DEGs, GSEA using Reactome did not yield enriched pathways. KEGG identified enrichment of ABC transporters, whereas GO analysis indicated associations with plasma membrane regions, homophilic cell adhesion via adhesion molecules, and extracellular matrix components (see Fig. 2).

We further investigated whether expression of any of the DEGs correlated with disease activity. Expression of nine genes correlated with the patients’ CAS at the time of decompression surgery (positive correlation: *SAMD3*, *LINC02863*, *TMEM132A*, *GRIK1-AS1*, *USP30-AS1*, *MMP13*, *IDH1-AS1*, and *CCNB2*; and negative correlation: *ARHGEF33*; see Fig. 2).

We next estimated the cellular composition of the tissue samples by computational deconvolution of the transcriptome data. Estimated proportions of 11 cell types (adipocytes, preadipocytes, fibroblasts, endothelial cells, perivascular cells, B cells, CD4+ T cells, CD8+ T cells, monocytes, neutrophils, and NK cells) revealed a higher abundance of B cells and neutrophils, and a lower abundance of preadipocytes in TED samples compared with controls (see Fig. 2). Given the limitations of the reference datasets used (prebuilt reference and public single-cell RNA-seq template not specific to retro-orbital fat tissue), we validated these findings by examining the expression of genes specific and unique to T cells (*CD3D/E*), cytotoxic T cells (*CD8A/B*), *FOXP3* regulatory T cells (*FOXP3*), B cells/plasma cells (*MS4A1*, *CD79B*, *IGHD*, and *IGHM*), neutrophils (*FCGR3B*), and monocytes/macrophages (*CD163*, *CD33*, and *MSR1*). Expression of genes specific to neutrophils and B cells/plasma cells was elevated in TED samples compared with controls, consistent with deconvolution results (see Fig. 3). In addition, we detected increased expression of genes specific for total T cells and cytotoxic T cells (see Fig. 3).

**Figure 4.**
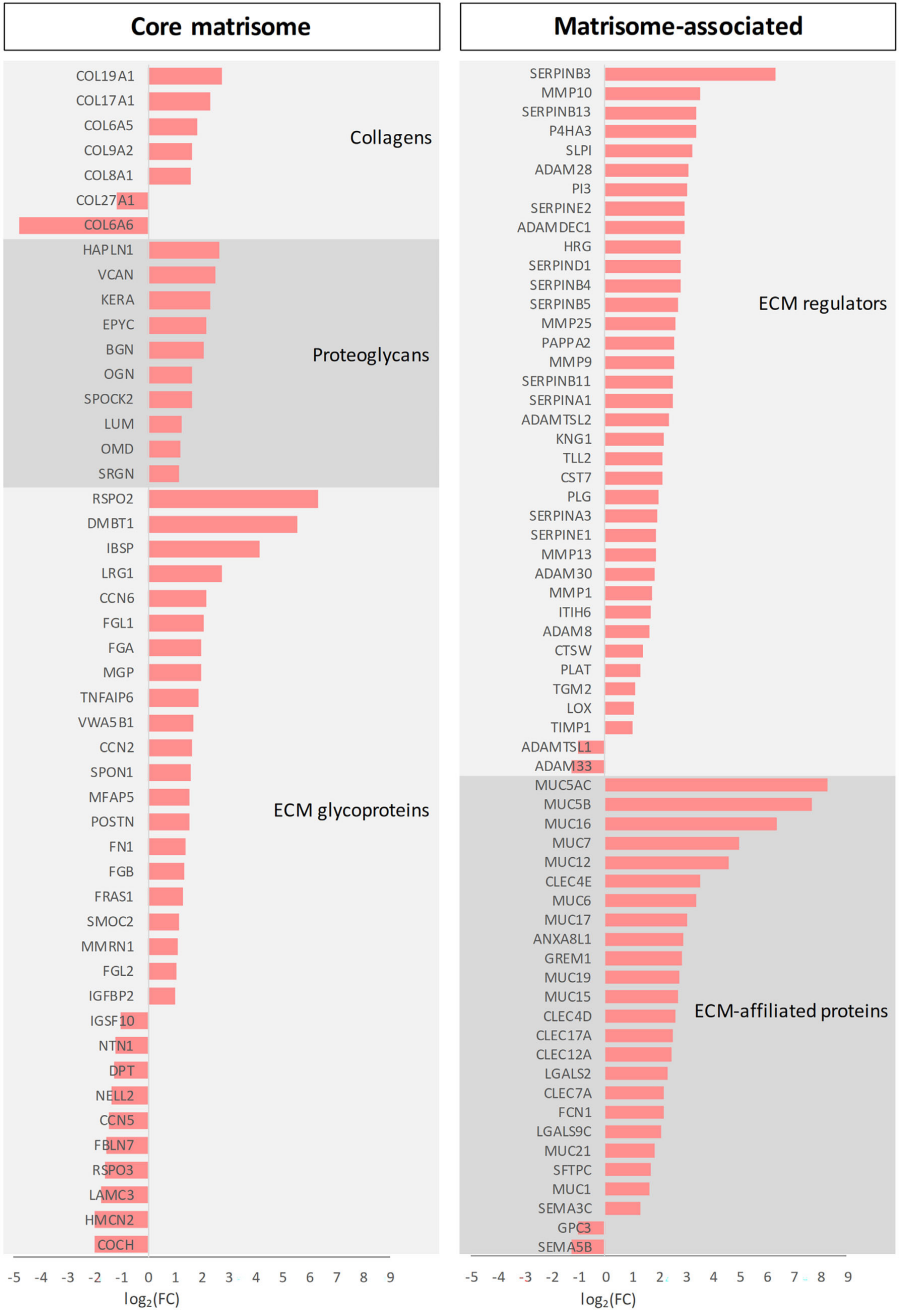
Differentially expressed genes (DEGs) encoding core matrisome components and matrisome-associated proteins identified in retro-orbital fat tissue from patients with TED. TED, thyroid eye disease.

As remodeling of the extracellular matrix (ECM) is a pathological hallmark of TED, we analyzed the expression of matrisome genes,^18^ encompassing both core ECM components (collagens, glycoproteins, and proteoglycans) and matrisome-associated proteins (Fig. 4). The most upregulated genes (log_2_FC > 3) included *MUC5AC*, *MUC5B*, *MUC16*, *SERPINB3*, *RSPO2*, *DMBT1*, *MUC7*, *MUC12*, *IBSP*, *MMP10*, *CLEC4E*, *SERPINB13*, *MUC6*, *P4HA3*, *SLPI*, *ADAM28*, *MUC17*, and *PI3*. The most downregulated genes (log_2_FC < −1.5) were *FBLN7*, *RSPO3*, *LAMC3*, *HMCN2*, *COCH*, and *COL6A6*.

### Transcriptome Analysis of Cells Obtained From Ocular Surface Wash

**Figure 5.**
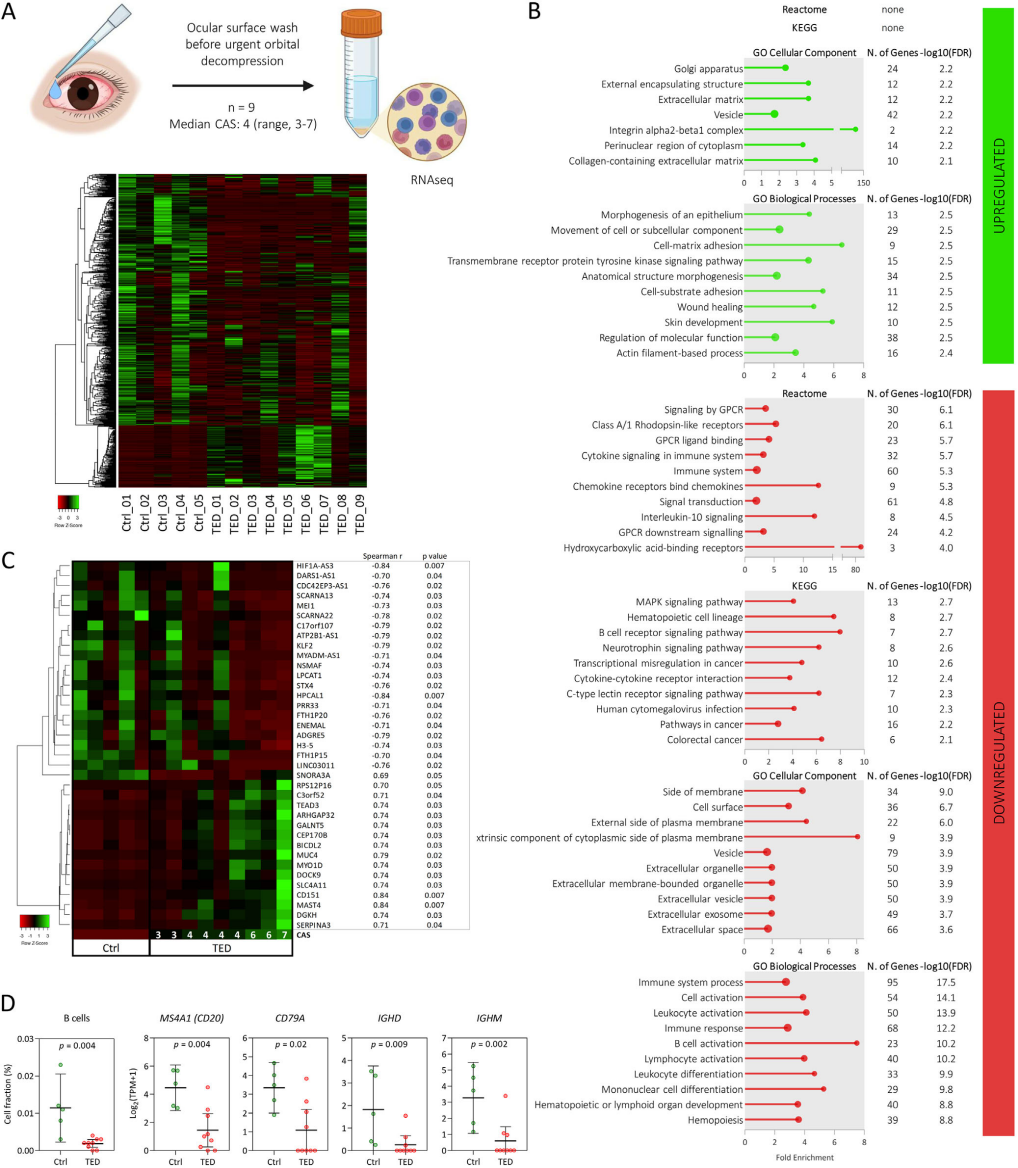
Transcriptomic profiling of ocular surface wash samples. (**A**) Heatmap of 715 differentially expressed genes (DEGs) identified in cells obtained from ocular surface wash from patients with active TED requiring urgent orbital decompression, compared to 5 control samples. The diagram at the top of panel **A** was created with BioRender.com. (**B**) Gene set enrichment analysis of DEGs using Reactome, KEGG, and GO and databases showing either all enriched pathways or the top 10 based on significance. (**C**) DEGs whose expression levels correlated with the patient's CAS at the time of surgery. (**D**) Estimated abundance of B cells in ocular surface wash samples, based on computational deconvolution of transcriptomic data, and expression of genes specific to B/plasma cells. CAS, clinical activity score; GO, Gene Ontology; KEGG, Kyoto Encyclopedia of Genes and Genomes; TED, thyroid eye disease.

To investigate gene expression changes in cells obtained from ocular surface wash from nine patients with active TED requiring urgent orbital decompression, RNA-seq was performed. Despite low cellularity in the majority of ocular surface wash samples, low RNA yield and quality, all samples fulfilled the input requirements for libraries preparation using SMARTer Stranded Total RNA-Seq Kit version 3 – Pico Input Mammalian with Unique Molecular Identifiers. A total of 715 genes were found to be differentially expressed in active TED compared with cells from ocular surface wash obtained from 5 control subjects using the cutoff criteria of *P* adjusted value < 0.05 and |log_2_FC| set to 1 (Supplementary Fig. S4). Of detected DEGs, 573 (80.1%) were downregulated and 142 (19.9%) upregulated in TED samples compared with controls (Fig. 5).

GSEA for DEGs in cells from ocular surface wash was performed using the Reactome, KEGG, and GO databases (see Fig. 5). Of upregulated DEGs, no enriched pathways were found in Reactome or KEGG. However, GO analysis revealed enrichment in cellular components such as the Golgi apparatus, ECM, and integrin α2β1 complex, and in biological processes including morphogenesis of an epithelium, cell-matrix adhesion, and wound healing. Among the top enriched Reactome pathways from downregulated DEGs were signaling by G-protein-coupled receptors, immune system, signal transduction, and IL-10 signaling. Top KEGG pathways included the MAPK signaling pathway, hematopoietic cell lineage, and B cell receptor signaling. GO enrichment analysis of downregulated DEGs revealed cell surface and extracellular vesicle among the top cellular components and B cell activation and immune system processes among the top biological processes.

Next, we looked at gene expression of targets of approved targeted therapy or targets currently being evaluated in clinical trials (*TSHR*, *IGF1R*, *MS4A1* [CD20], *IL6*, *IL6R*, *IL11*, *IL11RA*, and *FCGRT* [FcRn])^17^ in ocular surface wash samples. Of the genes analyzed, only upregulation of *IGF1R* was detected and *MS4A1* (CD20) was observed downregulated (Supplementary Fig. S5).

We further investigated whether expression of any of the DEGs in ocular surface wash samples correlated with disease activity. Expression of 37 genes correlated with the patients’ CAS at the time of decompression surgery (positive correlation: *SNORA3A*, *RPS12P16*, *C3orf52*, *TEAD3*, *ARHGAP32*, *GALNT5*, *CEP170B*, *BICDL2*, *MUC4*, *MYO1D*, *DOCK9*, *SLC4A11*, *CD151*, *MAST4*, *DGKH*, and *SERPINA3*; and negative correlation: *HIF1A-AS3*, *DARS1-AS1*, *CDC42EP3-AS1*, *SCARNA13*, *MEI1*, *SCARNA22*, *C17orf107*, *ATP2B1-AS1*, *KLF2*, *MYADM-AS1*, *NSMAF*, *LPCAT1*, *STX4*, *HPCAL1*, *PRR33*, *FTH1P20*, *ENEMAL*, *ADGRE5*, *H3-5*, *FTH1P15*, and *LINC03011*; see Fig. 5).

To estimate the cell composition in ocular surface wash samples, we performed computational deconvolution of the transcriptome data. Estimated proportions of 11 cell types (adipocytes, preadipocytes, fibroblasts, endothelial cells, perivascular cells, B cells, CD4+ T cells, CD8+ T cells, monocytes, neutrophils, and NK cells) revealed a lower abundance of B cells in TED samples compared with controls (see Fig. 5). Given the limitations of the reference datasets used (prebuilt reference and public single-cell RNA-seq template not specific to retro-orbital fat tissue), we validated these findings by examining the expression of genes specific and unique to T cells (*CD3D/E*), cytotoxic T cells (*CD8A/B*), *FOXP3*+ regulatory T cells (*FOXP3*), B cells/plasma cells (*MS4A1*, *CD79B*, *IGHD*, and *IGHM*), neutrophils (*FCGR3B*), and monocytes/macrophages (*CD163*, *CD33*, and *MSR1*). Only expression of genes specific to B cells/plasma cells was decreased in TED samples compared with controls, consistent with deconvolution results (see Fig. 5).

## Discussion

This study advances the understanding of TED pathogenesis through a unique transcriptomic analysis of retro-orbital fat tissue and cells obtained from ocular surface wash from patients with active, moderate-to-severe TED who required urgent orbital decompression surgery following standard IVGC therapy. First, it investigates samples obtained during the active disease stage from patients with moderate-to-severe TED (CAS = 3–7) requiring urgent decompression, capturing the molecular features of clinically aggressive disease. Second, it simultaneously analyzes both retro-orbital fat and ocular surface wash samples, enabling characterization of compartment-specific alterations for the first time.

This real-world study from referral center on cohort of patients with active, moderate-to-severe TED requiring therapy, identified one fifth of patients who needed urgent orbital decompression during the active disease phase. Compared with other patients, this subset presented with higher baseline CAS, elevated TSI levels, increased circulating neutrophil and monocyte counts, and prolonged time to disease inactivation (median 21 vs. 7 months) defined as the time from therapy initiation to reaching CAS 0. These data underscore the existence of a distinct high-risk clinical phenotype that may benefit from early identification and tailored immunomodulatory strategies.

Transcriptomic analysis of TED retro-orbital fat showed that the majority (86%) of DEGs were upregulated compared with control samples. Based on GSEA in biological processes, the largest subset of DEGs mapped to the immune system. A pronounced neutrophilic signature was observed, particularly in pathways related to neutrophil degranulation and extracellular trap formation. This was consistent with the higher abundance of neutrophils detected by computational deconvolution compared with control samples and with the increased circulating neutrophil counts in patients requiring urgent decompression compared with other patients with TED. These results are in line with recent observations reporting elevated circulating neutrophil levels in patients with active TED^19^^,^^20^ and demonstrate that neutrophil-driven inflammation plays a significant role in TED pathogenesis.

Beyond elevated neutrophil levels, a higher abundance of B cells and a lower abundance of preadipocytes in TED tissues was observed compared with control samples by computational deconvolution of the transcriptome data. In addition, increased expression of genes specific and unique for total T cells (*CD3*), cytotoxic T cells (*CD8*), and FOXP3 regulatory T cells (*FOXP3*) was detected in TED tissues compared with control samples. This aligns with a previous report of a correlation between T and B cells infiltrating retro-orbital fat tissues and TED activity^21^ and a recent report demonstrating enriched TED-specific clonal expansions in CD8 effector T cells that exhibited signs of enhanced T cell chemotaxis, exhaustion, and exerted potential pathogenic effects on fibrosis based on scRNA analysis of circulating immune populations.^22^

As autoreactive B cells producing autoantibodies against TSHR are a major pathogenetic feature of autoimmune thyroiditis and TED,^23^ we expectedly detected increased B cell abundance and upregulated expression of immunoglobulin genes in TED tissues compared with controls, further supporting the rationale for anti-CD20 therapy in TED. Notably, the observed upregulation of B cells in retro-orbital fat but downregulation in ocular surface wash in TED samples suggests differential compartment-specific immune activity.

Regarding ECM remodeling, a pathological hallmark of TED, this study provides a molecular atlas of ECM changes associated with active TED revealed by matrisome profiling.^18^ Strong upregulation of mucin genes (*MUC5AC*, *MUC5B*, *MUC16*, *MUC7*, *MUC12*, *MUC6*, and *MUC17*) and ECM remodeling genes (e.g. *MMP10*, *P4HA3*, and *ADAM28*) were observed in TED tissue.

In contrast to retro-orbital fat, ocular surface wash samples exhibited a predominantly downregulated immune signature when compared with control samples. The expression of several transcripts correlated with patients’ disease activity (CAS), suggesting potential use as noninvasive biomarkers. The top correlating genes were *CD151*, *MAST4* (positive correlation), and *HPCAL1* (negative correlation). Although the role of these genes/protein products in autoimmune conditions is unclear, the tetraspanin CD151 is involved in various cellular processes, including cell-cell junction, signal transduction, epithelial-mesenchymal transition, angiogenesis, and exosome regulation^24^; MAST4 is a microtubule-associated kinase involved in PTEN signaling pathway^25^^,^^26^; and visinin-like protein 3 (VILIP-3) encoded by *HPCAL1* is a calcium-binding protein that regulates signal transduction in the brain and retina.^27^ Future studies should validate changes in ocular surface transcriptomics as predictors of active TED requiring urgent decompression.

In silico drug-gene interaction analysis has suggested several novel potential therapies, including the JAK inhibitor ruxolitinib, the BTK inhibitor (BTKi) ibrutinib or interleukin 2 (IL-2) in addition to currently used drugs according to the EUGOGO clinical guidelines (prednisone/prednisolone, rituximab, mycophenolic acid, and cyclosporine) and agents used in the treatment of other autoimmune diseases (cyclophosphamide, indomethacin, and methotrexate). Ruxolitinib is approved for the treatment of myelofibrosis, polycythemia vera, steroid-refractory graft-versus-host disease (GVHD), atopic dermatitis, and vitiligo.^28^ Given the established benefit of interleukin 6 (IL-6) receptor blockade by tocilizumab in TED and the role of JAK as a downstream mediator of IL-6 signaling, JAK inhibition may deserve consideration in future studies in TED. Similarly, BTKi, which blocks BTK, an essential component of the B cell receptor pathway whose signaling is indispensable for B cell survival and activation, and which are approved for B cell malignancies and chronic GVHD,^29^ may provide an alternative strategy to suppress pathogenic B cell activity in TED. Consistent with this rationale, targeting B cells with anti-CD20 rituximab has already shown clinical benefit in TED management. Regarding IL-2, a recent clinical trial demonstrated clinical improvement in 13 autoimmune diseases with low doses of IL-2, which can specifically activate regulatory T cells.^30^

The strength of this study is the cohort of real-world patients requiring urgent decompression in the active disease stage, enabling insight into pathogenesis of TED that are under-represented in the current literature. Moreover, the integration of transcriptomic data from both retro-orbital fat tissue and a minimally invasive sampling method of ocular surface wash provides a multifaceted view of TED. Limitations include the modest sample size, and future studies should validate our findings in larger cohorts or complement them with functional validation. Additionally, because patients were receiving IVGC therapy prior urgent decompression according to clinical guidelines,^6^ treatment effects on gene expression cannot be excluded.

In summary, this study performed a comprehensive transcriptomic analysis of retro-orbital fat from patients with active TED requiring urgent decompression and demonstrated that the majority of differentially expressed genes were upregulated compared with control samples, with the largest subset of these genes mapping to the immune system. Novel insights into TED pathogenesis were gained, notably the identification of neutrophils as active participants in the pathogenesis of TED. Additionally, the study provides a detailed molecular atlas of immune and matrisome alterations in retro-orbital fat tissue associated with TED and confirms elevated infiltration of B and T cells in affected tissue. Furthermore, it nominates gene expression markers from noninvasive ocular surface wash sampling that correlate with disease activity and suggest new candidate drugs, including JAK and BTK inhibitors, which may broaden the therapeutic options in TED.

## Supplementary Material

Supplement 1

Supplement 2
