# Supplementary material for: Transcriptomic Profiling of Orbital Fat Tissue and Ocular Surface Wash in Active Thyroid Eye Disease Requiring Urgent Orbital Decompression
Source: Invest Ophthalmol Vis Sci. 2025 Dec 29;66(15):71. doi: 10.1167/iovs.66.15.71 (PMC12758420; doi:10.1167/iovs.66.15.71)
Supplement: Supplement 1 [file iovs-66-15-71_s001.docx]

The Supplementary File

**Transcriptomic profiling of orbital fat tissue and ocular surface wash in active thyroid eye disease requiring urgent orbital decompression**

Anna Petrackova et al.

**Contents:**

**Table S1:** RNA sample quality and quantity.

**Table S2**: RNA-seq quality metrics.

**Table S3:** Raw gene read counts, TPM values and differentially expressed genes. (Please see the "Supplementary Table S3.xlsx" file.)

**Table S4:** Cell-type specific and unique genes for T cells, B/plasma cells, neutrophils, monocytes/macrophages.

**Table S5:** Clinical characteristics of the study cohort.

**Figure S1:** Principal component analysis (PCA) of RNA-seq samples from (A) orbital fat tissue and (B) ocular surface wash.

**Figure S2:** Volcano plot showing differentially expressed genes in TED tissues compared to control tissues using the cut-off criteria of p adjusted value < 0.05 and |log2FC| set to 1.

**Figure S3**: Gene expression of targets of approved targeted therapy or targets currently being evaluated in clinical trials: *TSHR, IGF1R, MS4A1* (CD20), *IL6, IL6R, IL11, IL11RA, FCGRT* (FcRn) in retro-orbital tissue samples.

**Figure S4:** Volcano plot showing differentially expressed genes in TED ocular surface wash samples compared to control samples using the cut-off criteria of p adjusted value < 0.05 and |log2FC| set to 1.

**Figure S5:** Gene expression of targets of approved targeted therapy or targets currently being evaluated in clinical trials: *TSHR, IGF1R, MS4A1* (CD20), *IL6, IL6R, IL11, IL11RA, FCGRT* (FcRn) in ocular surface wash samples.

**Supplementary Table S1:** RNA sample quality and quantity.

| Specimen | Group | Sample | Concentration (ng/µl) | RIN |
| --- | --- | --- | --- | --- |
| fat tissue | control | Ctrl_1 | 483 | 6 |
| fat tissue | control | Ctrl_2 | 621 | 7.3 |
| fat tissue | control | Ctrl_3 | 1068 | 5.3 |
| fat tissue | control | Ctrl_4 | 789 | 7.3 |
| fat tissue | control | Ctrl_5 | 826 | 7.1 |
| fat tissue | TED | TED_1 | 860 | 7.3 |
| fat tissue | TED | TED_2 | 12.3 | 7.1 |
| fat tissue | TED | TED_3 | 33.4 | 6.4 |
| fat tissue | TED | TED_4 | 25.1 | 7.6 |
| fat tissue | TED | TED_5 | 37.0 | 5.5 |
| fat tissue | TED | TED_6 | 447 | 4.6 |
| fat tissue | TED | TED_7 | 1127 | 7.6 |
| fat tissue | TED | TED_8 | 167 | 5.9 |
| fat tissue | TED | TED_9 | 1112 | 7.3 |
| fat tissue | TED | TED_10 | 318 | 7.2 |
| fat tissue | TED | TED_11 | 46.3 | 8.4 |
| fat tissue | TED | TED_12 | 37.8 | 9.6 |
| fat tissue | TED | TED_13 | 26.6 | 7.0 |
| ocular surface wash | control | Ctrl_1 | 147 | 3.2 |
| ocular surface wash | control | Ctrl_2 | 3.9 | 2.8 |
| ocular surface wash | control | Ctrl_3 | 4.0 | 4.1 |
| ocular surface wash | control | Ctrl_4 | 91.2 | 4.6 |
| ocular surface wash | control | Ctrl_5 | 4.0 | 2.6 |
| ocular surface wash | TED | TED_1 | 12.2 | 2.7 |
| ocular surface wash | TED | TED_2 | 17.9 | 6.8 |
| ocular surface wash | TED | TED_3 | 11.0 | 2.7 |
| ocular surface wash | TED | TED_4 | 3.6 | 2.5 |
| ocular surface wash | TED | TED_5 | 0.3 | 3.2 |
| ocular surface wash | TED | TED_6 | 4.4 | 2.6 |
| ocular surface wash | TED | TED_7 | 0.3 | 2.7 |
| ocular surface wash | TED | TED_8 | 0.5 | 3.1 |
| ocular surface wash | TED | TED_9 | 0.4 | 2.8 |

**Supplementary Table S2**: RNA-seq quality metrics.

| Specimen | Group | Sample | M PE reads in raw fastq | M Reads mapped in bam file (samtools flagstat) | Aligned (STAR %) | M aligned reads (uniquelly mapped + multimapped) | M uniquelly mapped reads | % processed reads that passed deduplication (UMI) |
| --- | --- | --- | --- | --- | --- | --- | --- | --- |
| fat tissue | control | Ctrl_1 | 31.663656 | 67.312077 | 71.04 | 22.49386122 | 17.91529656 | 82.25 |
| fat tissue | control | Ctrl_2 | 21.701873 | 44.91574 | 70.01 | 15.19348129 | 11.97726371 | 80.43 |
| fat tissue | control | Ctrl_3 | 32.908248 | 72.618469 | 75.85 | 24.96090611 | 20.01150561 | 80.06 |
| fat tissue | control | Ctrl_4 | 23.409533 | 48.605634 | 70.87 | 16.59033604 | 12.62476115 | 75.72 |
| fat tissue | control | Ctrl_5 | 19.692342 | 44.996646 | 78.42 | 15.4427346 | 12.41799087 | 82.47 |
| fat tissue | TED | TED_1 | 31.823424 | 61.707307 | 67.56 | 21.49990525 | 16.98734373 | 81.32 |
| fat tissue | TED | TED_2 | 29.09847 | 73.718605 | 69.68 | 20.2758139 | 13.36201742 | 76.05 |
| fat tissue | TED | TED_3 | 27.288633 | 68.233507 | 73.02 | 19.92615982 | 13.55699287 | 74.55 |
| fat tissue | TED | TED_6 | 17.986944 | 40.19146 | 64.3 | 11.56560499 | 8.34774071 | 83.65 |
| fat tissue | TED | TED_7 | 21.562257 | 44.475979 | 69.33 | 14.94911278 | 11.27921664 | 77.65 |
| fat tissue | TED | TED_8 | 34.153754 | 75.48566 | 75.33 | 25.72802289 | 19.36517852 | 77.38 |
| fat tissue | TED | TED_9 | 17.386345 | 37.375619 | 66.94 | 11.63841934 | 8.766195149 | 81.39 |
| fat tissue | TED | TED_10 | 18.593911 | 43.861728 | 72.56 | 13.49174182 | 10.29544852 | 84.04 |
| fat tissue | TED | TED_11 | 20.774779 | 44.833205 | 64.36 | 13.37064776 | 9.951119141 | 84.22 |
| fat tissue | TED | TED_12 | 23.719602 | 50.304213 | 66.99 | 15.88976138 | 12.27489404 | 83.61 |
| fat tissue | TED | TED_13 | 31.973967 | 65.455695 | 67.06 | 21.44174227 | 16.00616788 | 78.07 |
| ocular surface wash | control | Ctrl_1 | 35.026876 | 65.893585 | 66.6 | 23.32789942 | 18.73937866 | 81.7 |
| ocular surface wash | control | Ctrl_2 | 29.969552 | 46.137429 | 80.08 | 23.99961724 | 21.206455 | 69.87 |
| ocular surface wash | control | Ctrl_3 | 30.524274 | 36.038101 | 47.95 | 14.63638938 | 10.79948814 | 64.1 |
| ocular surface wash | control | Ctrl_4 | 39.915258 | 76.973246 | 78.36 | 31.27759617 | 26.86696016 | 82.12 |
| ocular surface wash | control | Ctrl_5 | 34.431638 | 12.345178 | 10.53 | 3.625651481 | 2.65467929 | 82.67 |
| ocular surface wash | TED | TED_1 | 37.853891 | 19.052033 | 20.38 | 7.714622986 | 6.510869252 | 78.21 |
| ocular surface wash | TED | TED_2 | 30.968683 | 3.908305 | 5.04 | 1.560821623 | 1.334750237 | 77.08 |
| ocular surface wash | TED | TED_3 | 23.424623 | 39.486237 | 78.47 | 18.38130167 | 16.40426349 | 78.8 |
| ocular surface wash | TED | TED_4 | 18.706055 | 31.455913 | 82.29 | 15.39321266 | 14.08378881 | 77.23 |
| ocular surface wash | TED | TED_5 | 19.840868 | 25.819675 | 71.67 | 14.2199501 | 12.74775769 | 66.54 |
| ocular surface wash | TED | TED_6 | 16.910625 | 25.610093 | 64.16 | 10.849857 | 8.835801563 | 68.67 |
| ocular surface wash | TED | TED_7 | 16.831217 | 12.247915 | 45.3 | 7.624541301 | 6.389129973 | 50.49 |
| ocular surface wash | TED | TED_8 | 20.38791 | 33.944652 | 64.48 | 13.14612437 | 11.61091474 | 87.86 |
| ocular surface wash | TED | TED_9 | 20.233991 | 26.083763 | 59.27 | 11.99268647 | 9.208489304 | 59.58 |

Abbreviations: PE: paired-end; UMI: unique molecular identifiers.

**Supplementary Table S3:** Raw gene read counts, TPM values and differentially expressed genes. (Please see the "Supplementary Table S3.xlsx" file.)

**Supplementary Table S4**: Cell-type specific and unique genes for T cells, B/plasma cells, neutrophils, monocytes/macrophages were defined by a Tau specificity score > 0.80 using The Human Protein Atlas database.

| Gene | Tau specificity score | Single cell type specificity | Single cell type expression cluster |
| --- | --- | --- | --- |
| CD8A | 0.87 | Cell type enriched (T-cells) | T-cells - T-cell receptor (mainly) |
| CD8B | 0.88 | Cell type enriched (T-cells) | NK-cells - Adaptive immune response (mainly) |
| CD3D | 0.84 | Cell type enriched (T-cells) | NK-cells & T-cells - Immune system & Transcription (mainly) |
| CD3E | 0.86 | Group enriched (T-cells, NK-cells) | T-cells - T-cell receptor (mainly) |
| FOXP3 | 0.95 | Cell type enriched (T-cells) | T-cells - T-cell receptor (mainly) |
| IL7R | 0.80 | Cell type enriched (T-cells) | NK-cells & T-cells - Immune system & Transcription (mainly) |
| MS4A1 | 0.94 | Cell type enriched (B-cells) | B-cells - B-cell function (mainly) |
| CD79B | 0.86 | Cell type enriched (B-cells) | B-cells - B-cell function (mainly) |
| IGHD | 0.95 | Group enriched (Plasma cells, B-cells) | Plasma cells - Protein processing (mainly) |
| IGHM | 0.89 | Group enriched (Plasma cells, B-cells) | Plasma cells - Protein processing (mainly) |
| FCGR3B | 0.95 | Cell type enriched (Serous glandular cells) | Neutrophils - Degranulation (mainly) |
| CD163 | 0.84 | Group enriched (Kupffer cells, Macrophages) | Macrophages - Innate immune response (mainly) |
| CD33 | 0.87 | Group enriched (Kupffer cells, Hofbauer cells, Granulocytes, Macrophages, Langerhans cells, Monocytes) | Monocytes - Innate immune response (mainly) |
| MSR1 | 0.82 | Group enriched (Kupffer cells, Monocytes, Macrophages, Hofbauer cells, Langerhans cells) | Macrophages - Innate immune response (mainly) |

**Supplementary Table S5**: Clinical characteristics of the study cohort.

|  | Patients with TAO undergoing urgent orbital decompression (n=15) | Other patients with TAO (n=47)* |
| --- | --- | --- |
| Median age (years, min-max) | 55 (40-78) | 52 (21-78) |
| Female/Male | 8/7 (53%/47%) | 32/15 (68%/32%) |
| Smoking history (yes/no) | 8/7 (53%/47%) | 21/26 (45%/55%) |
| ivGC cumulative dose, g, median (min-max) | 6.0 (4.5-7.5) | 6.0 (1.5-7.5) |
| CAS, median (min-max) | 4.0 (3.0-6.0) | 3.0 (1.0-5.0) |
| WBC, 10^9^/L, median (min-max) | 9.5 (4.6-14.0) | 6.7 (4.3-12.9) |
| Neutrophil count, 10^9^/L, median (min-max) | 6.1 (3.5-10.5) | 3.9 (2.3-11.4) |
| Monocyte count, 10^9^/L, median (min-max) | 0.8 (0.3-1.0) | 0.6 (0.2-1.1) |
| Lymphocyte count, 10^9^/L, median (min-max) | 2.3 (0.7-3.2) | 2.1 (0.9-2.8) |
| TSH, mIU/L, median (min-max) | 0.02 (0.01-8.1) | 0.6 (0.01-104.4) |
| TSI, IU/L, median (min-max) | 9.3 (1.5-40.0) | 3.3 (0.1-40.0) |
| Anti-Tg, kU/L, median (min-max) | 1.3 (1.3-845.0) | 1.3 (1.0-1,000.0) |
| Anti-TPO, kU/L, median (min-max) | 42.7 (28-6,210.1) | 134.3 (1.0-13,000.0) |
| FT4, pmol/L, median (min-max) | 19.3 (10.1-29.8) | 15.2 (2.6-40.1) |
| FT3, pmol/L, median (min-max) | 5.3 (3.4-18.6) | 5.2 (2.0-28.8) |

* Excluding steroid-resistant patients.

Abbreviations: Anti-Tg: anti-thyroglobulin antibody; Anti-TPO: anti-thyroid peroxidase antibodies; CAS: clinical activity score; FT3: free triiodothyronine; FT4: free thyroxine; TAO: thyroid-associated orbitopathy; TSI: thyroid-stimulating immunoglobulins; TTID: time to inactive disease; WBC: white blood cell count.

**Figure S1:** Principal component analysis (PCA) of RNA-seq samples from (A) orbital fat tissue and (B) ocular surface wash.

| A) | 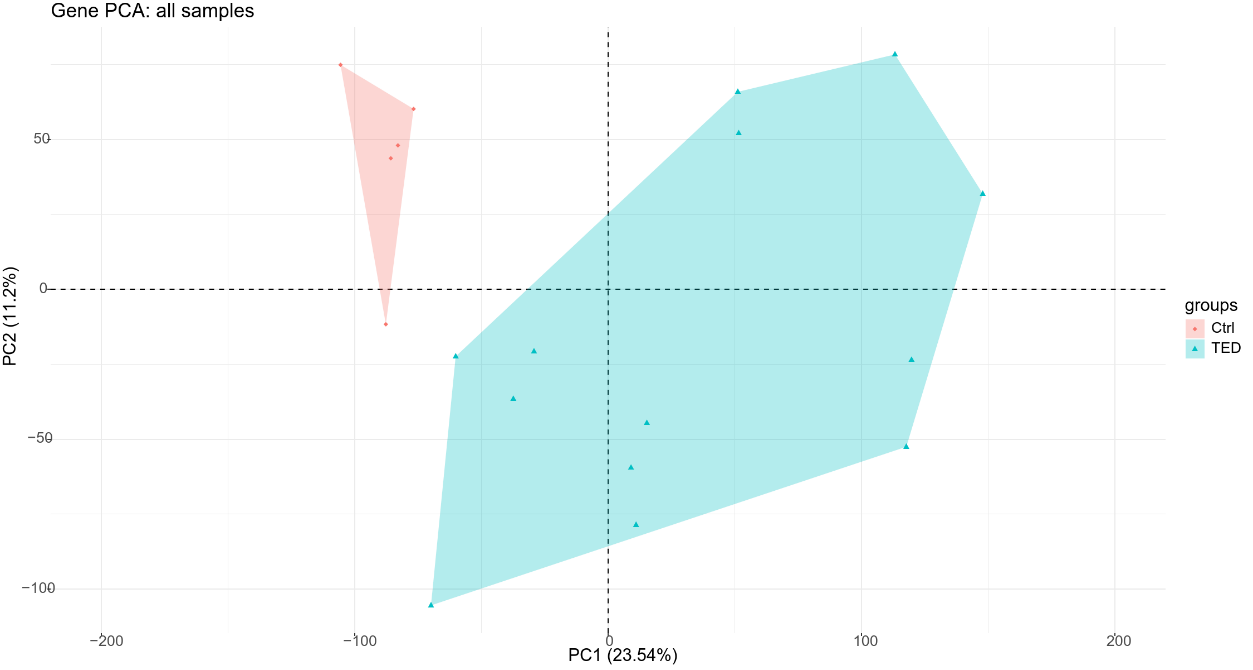 |
| --- | --- |
| B) | 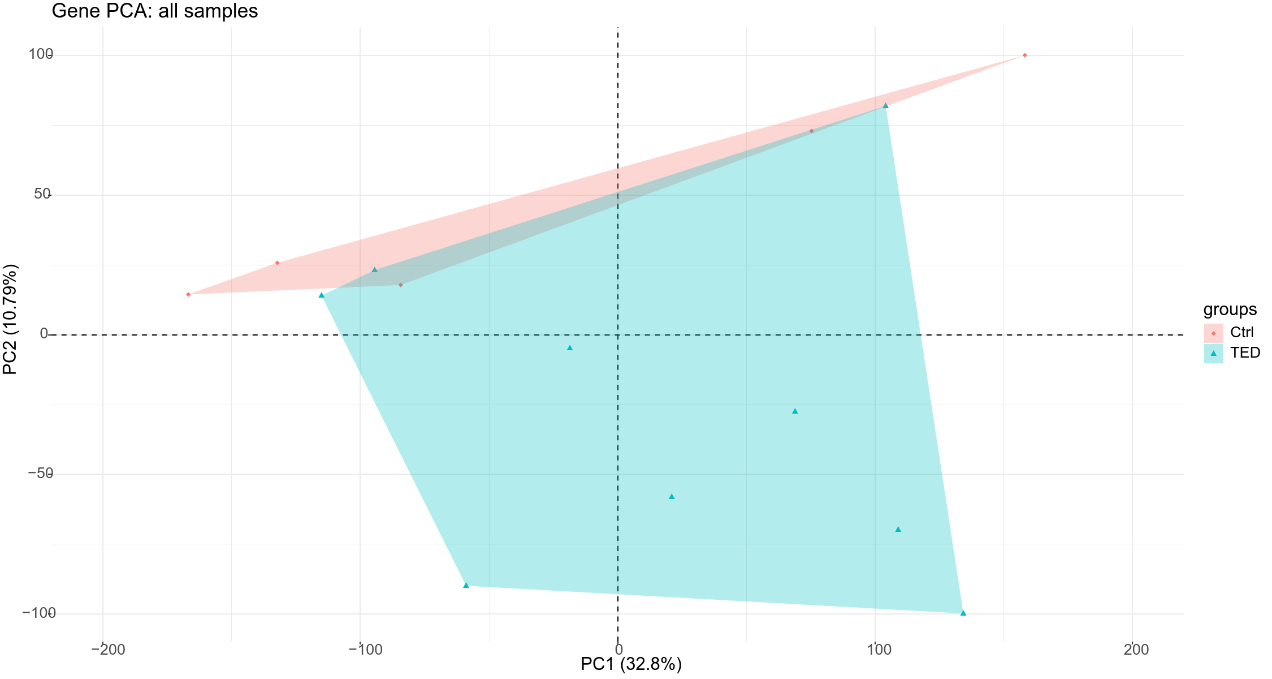 |

**Figure S2**: Volcano plot showing differentially expressed genes in TED tissues compared to control tissues using the cut-off criteria of p adjusted value < 0.05 and |log2FC| set to 1.


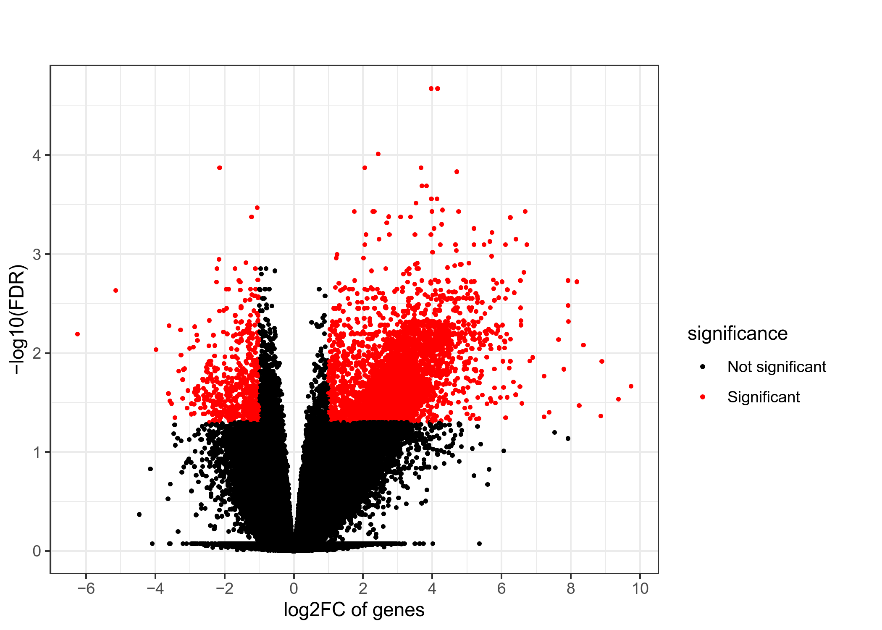


**Figure S3**: Gene expression of targets of approved targeted therapy or targets currently being evaluated in clinical trials: *TSHR, IGF1R, MS4A1* (CD20), *IL6, IL6R, IL11, IL11RA, FCGRT* (FcRn) in retro-orbital tissue samples.

**Figure S4:** Volcano plot showing differentially expressed genes in TED ocular surface wash samples compared to control samples using the cut-off criteria of p adjusted value < 0.05 and |log2FC| set to 1.


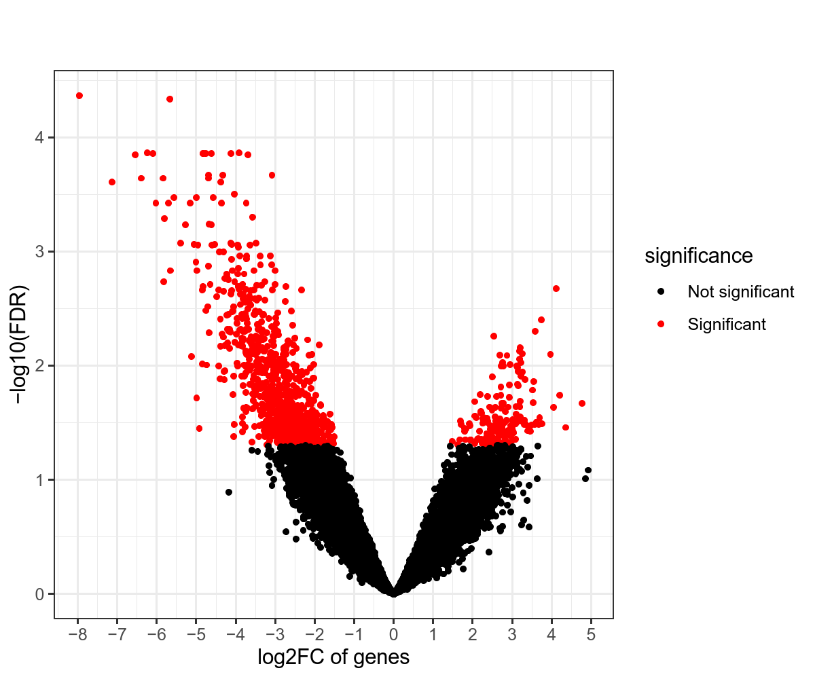


**Figure S5**: Gene expression of targets of approved targeted therapy or targets currently being evaluated in clinical trials: *TSHR, IGF1R, MS4A1* (CD20), *IL6, IL6R, IL11, IL11RA, FCGRT* (FcRn) in ocular surface wash samples.
